# Pharmaceutical Evaluation of Honokiol and Magnolol on Apoptosis and Migration Inhibition in Human Bladder Cancer Cells

**DOI:** 10.3389/fphar.2020.549338

**Published:** 2020-11-06

**Authors:** Hisao-Hsien Wang, Ying Chen, Chih-Ying Changchien, Hsin-Han Chang, Pei-Jyun Lu, Heidi Mariadas, Yu-Chen Cheng, Sheng-Tang Wu

**Affiliations:** ^1^ Division of Urology, Department of Surgery, Cheng Hsin General Hospital, Taipei, Taiwan; ^2^ Department of Biology and Anatomy, National Defense Medical Center, Taipei, Taiwan; ^3^ Department of General Medicine, Tri-Service General Hospital, National Defense Medical Center, Taipei, Taiwan; ^4^ Department of Medicine, National Defense Medical Center, Taipei, Taiwan; ^5^ Division of Urology, Department of Surgery, Tri-Service General Hospital, National Defense Medical Center, Taipei, Taiwan; ^6^ Department of Medical Planning, Medical Affairs Bureau Ministry of National Defense, Taipei, Taiwan

**Keywords:** honokiol, magnolol, apoptosis, autophagy, bladder cancer

## Abstract

Among herbal medicines, magnolia bark extract, particularly its components honokiol (Hono) and magnolol (Mag), has been widely documented to have antineoplastic properties. The present study aimed to evaluate the synergism of Hono and Mag in bladder cancer therapy both *in vitro* and *in vivo*. Treatment with Mag alone at concentrations up to 80 μM failed to have an antiproliferative effect. In contrast, the combination of Hono and Mag at 40 μM decreased viability, caused cell cycle arrest and enhanced the proportion of Annexin V/7AAD-positive cells. Moreover, Mag with Hono at 40 μM induced caspase 3-dependent apoptosis and autophagy. Neither Hono nor Mag alone had an anti-migratory effect on bladder cancer cells. In contrast, Hono and Mag at 20 μM inhibited the motility of TSGH8301 and T24 cells in wound-healing and Transwell assays. The above phenomena were further confirmed by decreased phosphorylated focal adhesion kinase (p-FAK), p-paxillin, integrin β1, and integrin β3 protein levels. In a nude mouse xenograft model, Mag/Hono administration preferentially retarded T24 tumor progression, which was consistent with the results of cellular experiments. Current findings suggest Hono and Mag treatment as a potential anticancer therapy for both low- and high-grade urothelial carcinoma.

## Introduction

According to World Health Organization (WHO) statistics, bladder cancer ranks as the sixth most common cancer worldwide, with approximately 430,000 new cases diagnosed each year (Burger et al., 2013). In Taiwan, bladder cancer is ninth most common cancer in men (Antoni et al., 2017). However, over the past 20 years, there has been little improvement in the diagnosis, therapy and 5-year survival rate of bladder cancer. Bladder cancer can be classified as non-muscle invasive bladder cancer, which affects an estimated 75–80% of bladder cancer patients and exhibits muscle invasiveness and frequent metastasis (DeGeorge et al., 2017). Primary treatment for non-muscle invasive bladder cancer is transurethral resection of the bladder tumor followed by intravesical mitomycin C chemotherapy. Unfortunately, tumor recurrence is observed in an estimated 65–70% of patients (DeGeorge et al., 2017). Therefore, drug development or the discovery of new pathogenic targets is the current goal of bladder cancer research.

The anticancer effects of honokiol (Hono) and magnolol (Mag) have been reported in various types of cancer cells. Hono caused apoptosis and blocks migration in lung cancers by ER stress and PI3K/AKT signaling (Zhang et al., 2019; Zhu et al., 2019). In addition, Hono induced autophagy in osteosarcoma through the ROS/ERK1/2 and PI3K/AKT cascades (Huang et al., 2018; Li et al., 2018). Autophagy in non-small lung cancer was also triggered by Mag treatment (Shen et al., 2017a). In addition, Mag was found to induce apoptosis through MAPK activation, ROS production, and mitochondrial-dependent pathways (Chen et al., 2019; Wen et al., 2019). The combined effects of Hono and Mag have been described in only human glioblastoma cells (Cheng et al., 2016). No study has focused on the effects of the combination of Hono and Mag in human bladder cancer cells.

Several reports report cell cycle arrest and migration inhibition in bladder cancer cells induced by Hono and Mag. Hono reduced the survival and migration of T24 cells through histone H3K27 methyltransferase (Zhang et al., 2015). In addition, Hono induced cell cycle arrest and ROS production in BFTC905 cells (Hsiao et al., 2019). Additionally, Hono inhibited invasion and epithelial-mesenchymal transition in J82 cells by reducing MMP9 and N-cadherin expression (Shen et al., 2017b). Furthermore, Mag caused cell cycle arrest of 5,637 human urinary cells at G2/M phase and extracellular signal-regulated kinase activation (Lee et al., 2008). Accordingly, the combination of Hono and Mag induced apoptosis and autophagy in human glioblastoma cells (Cheng et al., 2016). However, the synergistic effect of Hono and Mag on cell survival and migration in human bladder cancer cells and the involved signaling pathway remain unclear.

## Materials and Methods

### Human Bladder Cancer Cells

T24 and J82 cells were obtained from American Type Culture Collection. TSGH8301 cells were a generous gift from Dah-Shyong Yu (Division of Urology, Tri-Service General Hospital, National Defense Medical Center, Taiwan). The cells were grown in RPMI 1640 medium containing 10% fetal bovine serum and 100 IU/ml penicillin and streptomycin (pH 7.4) (all obtained from Thermo Fisher Scientific, Inc.) in a humidified atmosphere of 5% CO_2_ at 37°C.

### Drugs

Hono (Cat. No. HY-N0003, purity is 99.90%), Mag (Cat. No. HY-N0163, purity is 99.72%), and Ac-DEVD-CHO (Cat. No. HY-P1001, purity is 98.36%) were obtained from MedChemExpress (MCE, USA). 3-(4,5-Dimethyl-2-thiazolyl)-2,5-diphenyl-2H-tetrazolium bromide (MTT) and 3-methyladenine (3MA, Cat. No. M9281, purity ≥99%) was purchased from Sigma.

### MTT Assays

3 × 10^4^ bladder cancer cells were cultured in a 24-well plate. Hono, Mag, or both at different concentrations were then added to the cancer cells for 24 h. After the cells had been washed with phosphate-buffered saline (PBS) (137 mM NaCl, 2.7 mM KCl, 1.5 mM KH_2_PO_4_, 8 mM Na_2_HPO_4_, and pH 7.4), 0.5 mg/ml MTT was added, and the cells were incubated for another 3 h. The cells were then lyzed with DMSO (Sigma). The absorbance at 590 nm was measured.

### Flow Cytometric Analysis

A total of 5 × 10^5^ T24 or TSGH8301 bladder cancer cells were seeded in 6-well plates and attached for 24 h. For cell cycle analysis, cells were fixed with 70% EtOH and stained with PI solution. Annexin V-phycoerythrin and 7-aminoactinomycin D (7-AAD) (BD Biosciences) were processed for apoptosis analysis. After various drug treatments, the cells were harvested and stained with Annexin V according to the manufacturer’s instructions (BD Biosciences). The cells were then washed with PBS, stained with anti-Annexin V antibody and counterstained with 7AAD in binding buffer at room temperature for 15 min. The results were measured using a FACS Caliber flow cytometry analysis system (Becton Dickinson). Ten thousand cells in each sample were analyzed.

### Western Blotting

In brief, the cells were homogenized in mammalian protein extraction buffer (GE Healthcare) with a protease inhibitor cocktail (MCE) after the various treatments. Protein samples were electrophoresed on a 10% SDS-polyacrylamide gel and then transferred to a nitrocellulose membrane (Bio-Rad). Strips from the membrane were blocked with 5% non-fat milk in Tris-buffered saline, pH 7.4, containing 0.1% Tween (TBS-Tween). The strips were incubated with primary antibodies against phosphorylated-AKT (p-AKT)/AKT and p62 (Santa Cruz), integrin β1, β3, p-FAK/FAK, p-paxillin/paxillin (BD Biosciences), p-mTOR, mTOR, LC3β, and GAPDH (Cell Signaling) at 4°C overnight. After being washed, the strips were incubated with HRP-conjugated anti-rabbit or anti-mouse IgG antibodies diluted 1:5,000 (Cell Signaling). Next, the blots were incubated in ECL reagent for signal development (Bio-Rad). The densities of the bands on the nitrocellulose membrane were captured by SageCapture Microsoft Basic Application software and quantified by densitometry using Gel Pro 3.1 (Media Cybernetics), with the density of the control sample set to 100% and the density of each test sample expressed relative to the expression of the internal control. The phosphorylated proteins were normalized to total protein first.

### Wound-Healing Assay

Wound-healing migration assays were conducted by seeding 1 × 10^6^ cancer cells in 3.5-cm culture dishes to form a monolayer. The cells were then cultured for 8 h after being scratched with a P200 pipette tip and photographed; the migration assay results are representative of three different experiments and were analyzed with ImageJ.

### Transwell Assays

Transwell migration assays were prepared for by seeding cancer cells with 10% FBS culture medium in the upper chamber of a Transwell^®^ (Costar). As in Transwell assay, there is no difference between the exist of FBS or not in the upper chamber both in non-cancer (Omar Zaki et al., 2019) and cancer cells (Pijuan et al., 2019). Moreover, serum may confound the result while other chemoattractant is present. No matter the present of serum or not, the migration of cancer cells can be observed (Limame et al., 2012). After various treatment in 10% FBS culture medium and incubation at 37°C for 16 h, the cells on the lower side of the membrane were fixed with formalin and stained with Coomassie Brilliant Blue G250 (Sigma). For each experiment, the migrated cells in three randomly selected fields from each membrane were examined. Transwell invasion assays were prepared for by seeding cancer cells in the upper chamber of a Transwell. Before cell seeding, a coating buffer solution containing 3% Matrigel (BD Biosciences) was added to the upper chamber and then incubated for 2 h at 37°C. The cells on the lower side of the membrane were fixed with formalin and stained with Coomassie Brilliant Blue. For each experiment, the invaded cells in three randomly selected fields from each membrane were counted.

### *In Vivo* Xenograft Mouse Model

All mouse experiments were approved by the Laboratory Animal Center of the National Defense Medical Center, Taiwan (IACUC No. 19-229). The experimental animals were kept in the Laboratory Animal Center of National Defense Medical Center, and all experiments were carried out in a temperature-controlled (20–25°C) and 12 h light/dark cycle during the experimental period. BALB/cAnN.Cg-Foxn1nu/CrlNarl nude mice (20–25 g) were anesthetized with an O_2_/isoflurane mixture. T24-Luc2 cells were derived from the stable transfection of pLuc2-iRFP and selected with a BD FACSAria sorter with BD FACSDiva software (BD Biosciences). The cells were grown in RPMI 1640 medium containing 10% fetal bovine serum (pH 7.4) (Gibco) in a humidified atmosphere of 5% CO_2_ at 37°C. A total of 1 × 10^6^ T24 cells (mixed with Matrigel 1:1) were implanted into each mouse by subcutaneous injection and allowed to form into tumors for 1 month. The animals were divided into four groups (*N* = 8 each group) and received an ip injection of a control, Hono (25 mg/kg), Mag (25 mg/kg), or Hono + Mag (25 mg/kg for each drug). The bioluminescence intensities of the implanted tumors were monitored with a noninvasive In Vivo Imaging System (IVIS) every 3 days. The body weights of the mice were also measured while IVIS imaging was performed. After 20 days, the animals were sacrificed, and the tumors were imaged and homogenized for Western blotting.

### Statistical Analysis

All experiments were performed at least three times, and the results are expressed as the means ± SEMs for the total number of experiments. Differences between means were assessed using the Kruskal-Wallis test. The Mann-Whitney test was used for post hoc analysis. Statistical significance was set at *p* < 0.05.

## Results

### Honokiol and Magnolol Reduced Cell Survival in Bladder Cancer Cells

**FIGURE 1:**
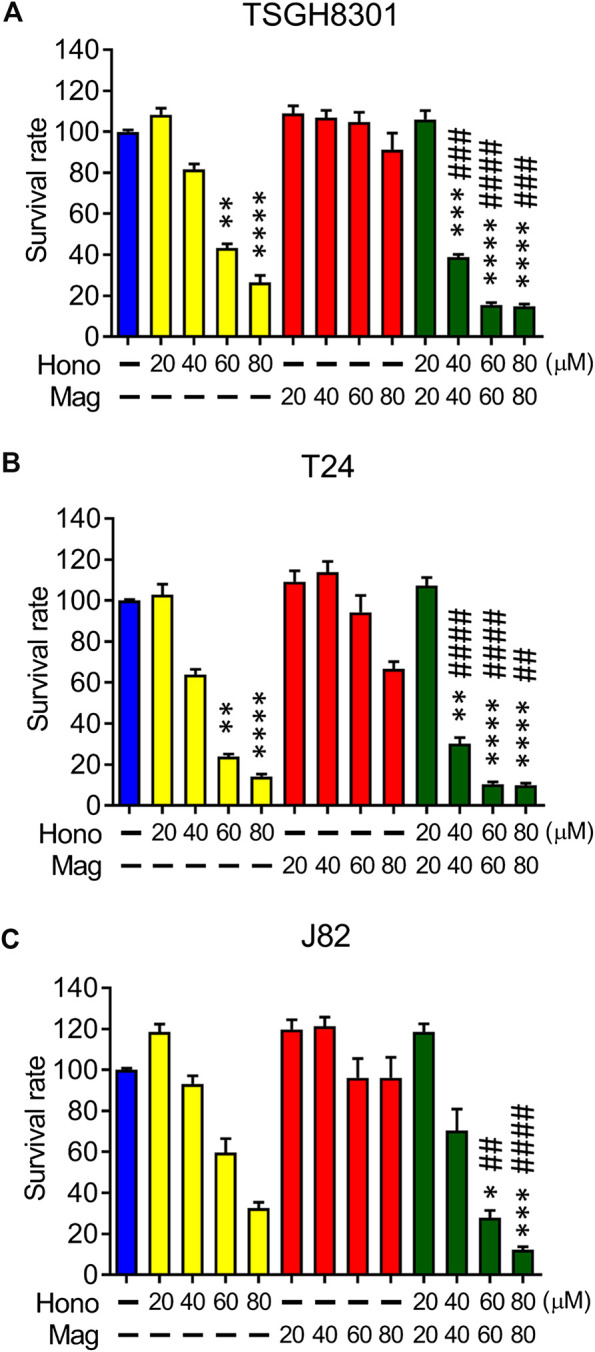
The effects of Hono, Mag, and Hono-Mag combination treatment on cell viability. **(A)** TSGH8301, **(B)** T24, and **(C)** J82 bladder cancer cells were treated with DMSO or 20, 40, 60, or 80 μM Hono, Mag, or Hono/Mag in combination for 24 h. After treatment, the survival rates were analyzed using MTT assays. *p < 0.05; **p < 0.01; ***p < 0.001 compared to the control group. ^#^ *p* < 0.05; ^##^ *p* < 0.01; ^###^ *p* < 0.001 compared to the group treated with the same concentration of Mag.

The effects of Hono and Mag on human bladder cancer cell survival were examined by MTT assay. Hono (60 μM) reduced cell survival to 43% and 24% in TSGH8301 and T24 cells, respectively (Figures 1A,B). In addition, Hono (80 μM) reduced cell survival to 27% and 14% in TSGH8301 and T24 cells, respectively (Figures 1A,B). In contrast, the survival rates of TSGH8301, T24, and J82 cells were unaffected by 20–80 μM Mag treatment (Figures 1A–C **)**. However, the combination of Hono and Mag from 20 to 80 μM significantly reduced the survival rate in both TSGH8301 and T24 cells to 30% (Figures 1A,B).

### Honokiol and Magnolol Inhibited the Cell Cycle and Induced Apoptosis and Autophagy

**FIGURE 2:**
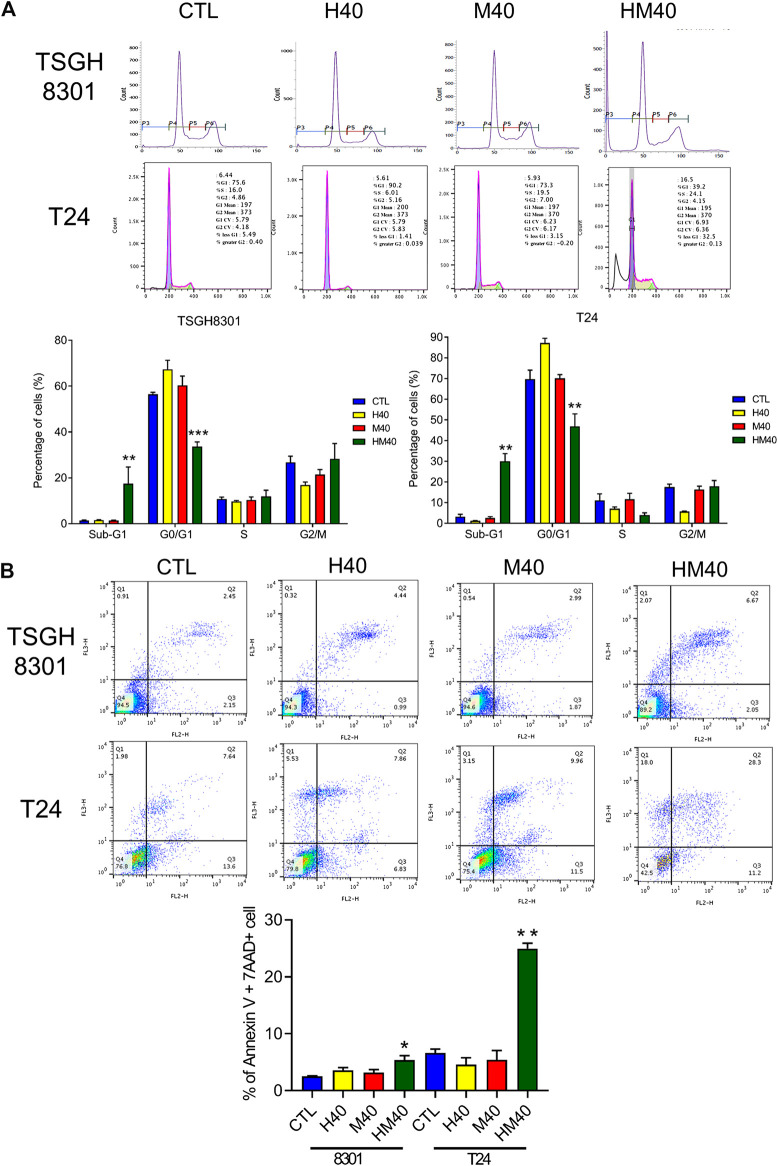
Cell cycle regulation and apoptosis induction by Hono, Mag, and Hono-Mag combination treatment in human bladder cancer cells. **(A)** TSGH8301 and T24 bladder cancer cells were treated with Hono, Mag, or Hono/Mag (40 μM) for 24 h. Cell cycle expression was then analyzed by FACS. Quantitative analyses of the populations of cells in difference phases of the cell cycle were conducted using BD FACSuite analysis software, and the results are shown in the lower panel. **(B)** TSGH8301 and T24 bladder cancer cells were treated with Hono, Mag, or Hono/Mag (40 μM) for 24 h. The proportions of apoptotic cells were determined using Annexin V/7AAD staining and flow cytometry analysis. The lower panel shows the percentage of apoptotic cells. *p < 0.05; **p < 0.01; ***p < 0.001 compared to the control group.

**FIGURE 3:**
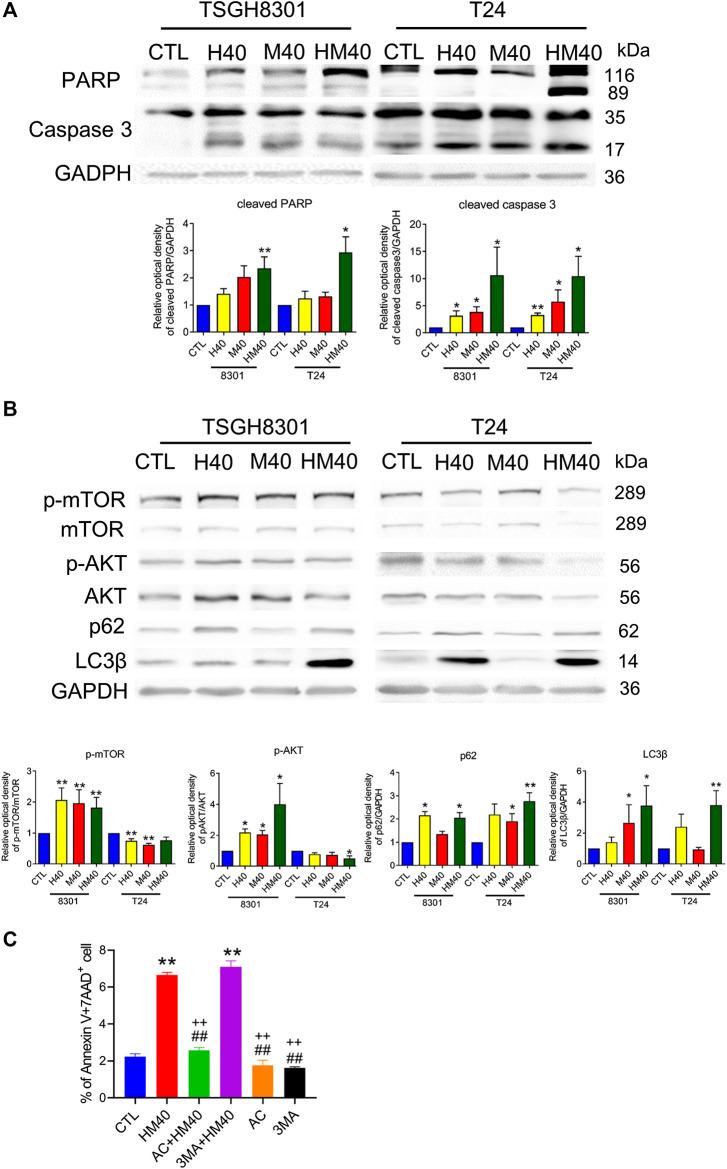
Hono-Mag promoted apoptosis and autophagy-associated proteins in bladder cancer cells. TSGH8301 and T24 bladder cells were treated with Hono, Mag, or Hono/Mag (40 μM) for 24 h. **(A)** The cell lysates were analyzed for the apoptotic-related proteins cleaved caspase 3 and cleaved PARP by Western blotting. **(B)** The cell lysates were analyzed for the autophagy-associated proteins p-AKT, AKT, p62, and LC3β. GAPDH was used as the loading control. The lower panels show the results of quantitative protein analyses. **(C)** TSGH8301 bladder cancer cells were treated with Hono/Mag (40 μM), Ac-DEVD-CHO (AC) + Hono/Mag, 3 MA + Hono/Mag, AC, or 3 MA for 24 h. The proportions of apoptotic cells were determined using Annexin V/7AAD staining and flow cytometry analysis. *p < 0.05; **p < 0.01 compared to the control group. ^##^ *p* < 0.01 compared to the Hono/Mag group. ^++^ *p* < 0.01 compared to the 3 MA + Hono/Mag group.

Next, the synergistic effect of Hono and Mag on the cell cycle was analyzed in TSGH8301 and T24 cells. As shown in Figure 2A, the HM40 group (40 μM Hono + 40 μM Mag) exhibited a significantly reduced G0/G1 phase cell population compared to the CTL, H40 (40 μM Hono), and M40 (40 μM Mag) groups of both bladder cancer cell lines. Moreover, the sub-G1 cell population was increased in the HM40 group (Figure 2A). These results showed that combined treatment with Hono and Mag caused cell cycle arrest and apoptosis in TSGH8301 and T24 cells. As sub-G1 cells were detected in the group subjected to combined treatment with Hono and Mag (Figure 2A), apoptosis was further confirmed by Annexin V and 7AAD staining. As expected, HM40 increased apoptosis by 5% and 25% in TSGH8301 and T24 cells, respectively (Figure 2B). In addition, cleaved caspase 3 and cleaved PARP levels were elevated by HM40 treatment (Figure 3A), indicating caspase-dependent apoptosis.

The mTOR pathway and autophagy are involved in the anticancer effects of Hono and Mag in human glioblastoma cells and ovarian carcinoma (Cheng et al., 2016; Lee et al., 2019). HM40 significantly elevated p-mTOR and p-AKT expression in TSGH8301 cells; in contrast, p-mTOR and p-AKT expression was decreased in T24 cells in the HM40 group (Figure 3B). Furthermore, HM40 strongly increased p62 and LC3β expression in both bladder cancer cell lines (Figure 3B). These results indicated the involvement of mTOR and autophagy in the effects of combined treatment with Hono and Mag on human bladder cancer cells.

Caspase 3 and autophagy inhibitors were used to rescue HM40-induced apoptosis. Interestingly, only caspase 3 inhibitor, Ac-DEVD-CHO blocked HM40-caused apoptosis (Figure 3C). Autophagy inhibitor, 3MA, could not reverse the apoptosis effect induced by HM40 treatment (Figure 3C). Therefore, the HM40-induced apoptotic effect was autophagy independent.

### Honokiol and Magnolol Blocked Migration by Downregulating the Focal Adhesion Complex

**FIGURE 4:**
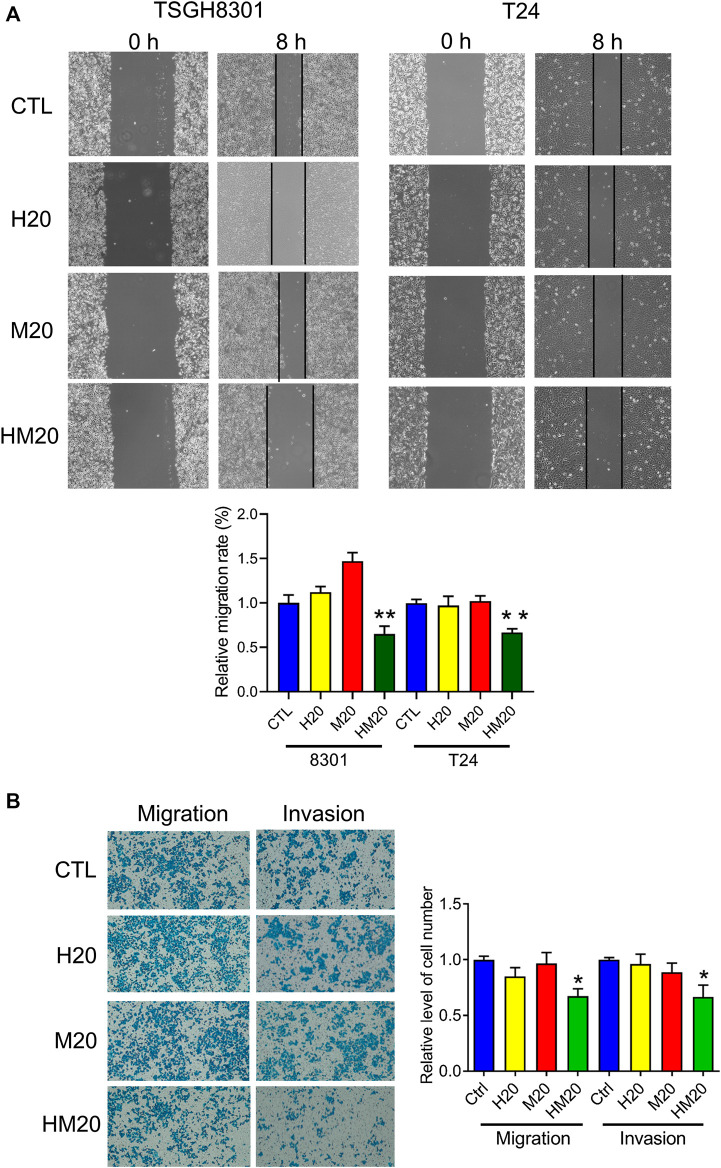
The effect of Hono-Mag on cell migration. **(A)** Confluent bladder cancer cells were scratched and incubated with Hono, Mag, or Hono/Mag (20 μM) for 8 h. The wound area was analyzed by ImageJ software and is expressed relative to that at 0 h. **(B)** T24 bladder cancer cells were seeded in the upper chambers of Transwells with or without Matrigel. After 24 h of incubation with Hono, Mag, or Hono/Mag (20 μM), cells in the lower chamber were counted. *p < 0.05; **p < 0.01 compared to the control group.

A wound-healing migration assay was performed to investigate the migratory abilities of human bladder cancer cells treated with Hono and Mag. Both Hono and Mag were used at 20 μM to avoid cell death. After 8 h of drug treatment, wound closure was significantly decreased in the HM20 group (Figure 4A), indicating that HM20 reduced the migration of TSGH8301 and T24 cells. Since TSGH8301 cells were undetectable in the Transwell assay, only T24 cells were used in the Transwell migration and invasion assays. The Transwell assay showed a decrease in the migratory and invasive abilities of T24 cells in the HM20 group (Figure 4B).

**FIGURE 5:**
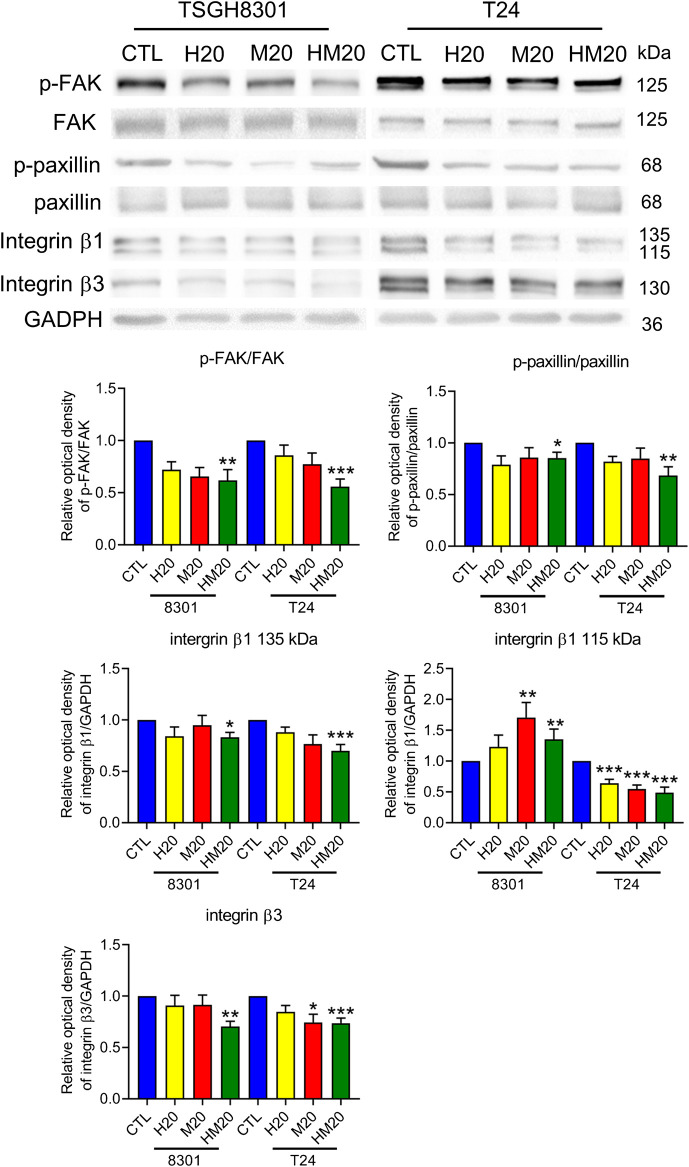
Hono-Mag reduced the activity of the focal adhesion complex. Bladder cancer cells were treated with Hono, Mag, or Hono/Mag (20 μM) for 24 h. Then, cell lysates were analyzed for integrin β1, FAK, p-FAK, paxillin, and p-paxillin by Western blotting. The results are representative of those obtained in five experiments. GAPDH was used as the loading control. Lower panels, quantitative analyses of the levels of the aforementioned proteins; *p < 0.05 compared to the control group.

Due to the inhibitory effect of combination treatment with Hono and Mag on migration, expression of the focal adhesive complex was investigated. As expected, p-FAK, p-paxillin, integrin β1, and integrin β3 levels were significantly decreased in the HM20 group (Figure 5). These results showed that the combination of Hono and Mag reduced human bladder cancer cell migration through downregulation of the focal adhesive complex.

### The Effect of Honokiol and Magnolol on Tumor Progression in a Xenograft Mouse Model

**FIGURE 6:**
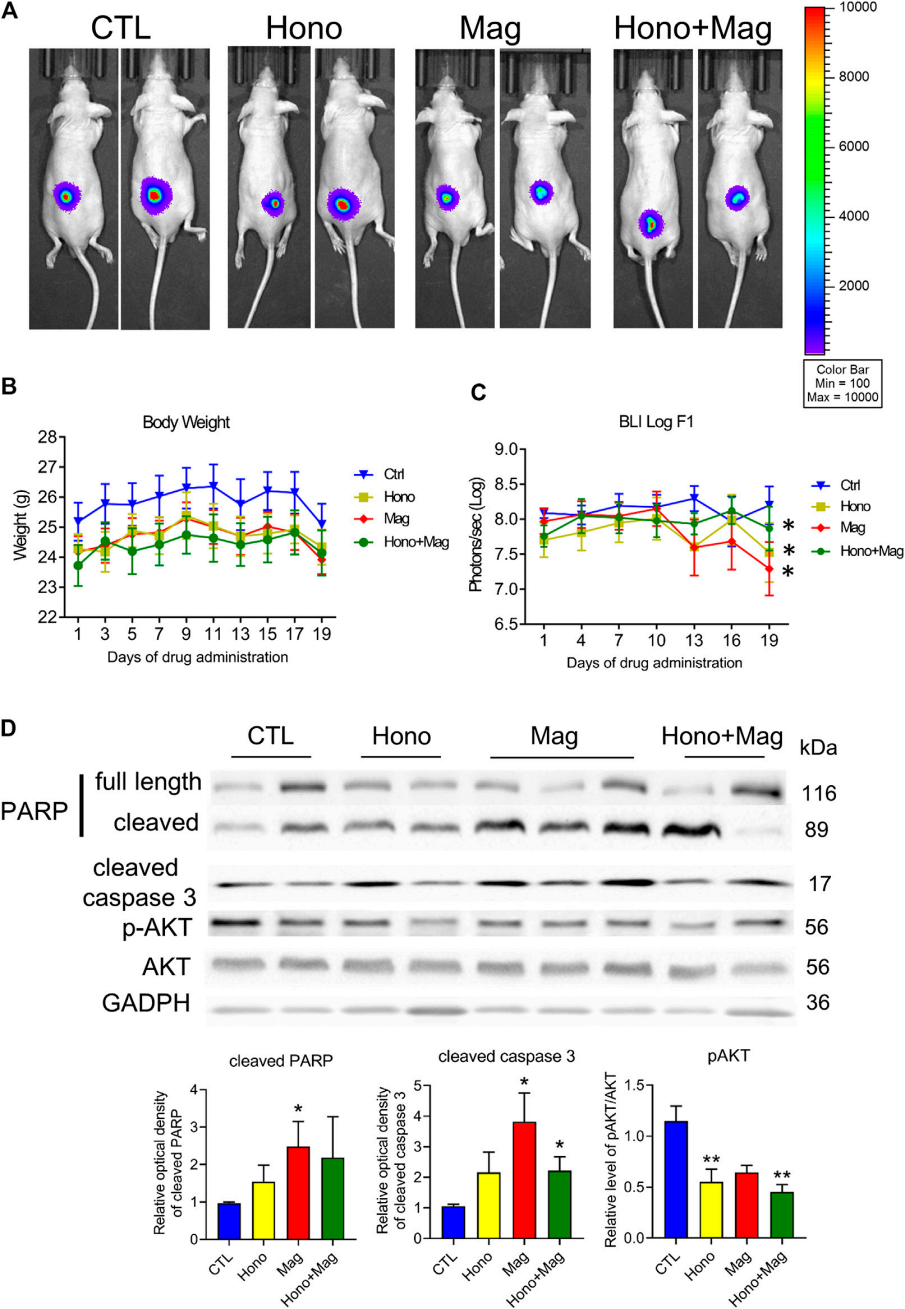
Hono and Mag treatment reduced tumor progression in human T24 bladder cancer cell xenograft model mice. **(A)** The *in vivo* bioluminescent imaging data from different groups were analyzed via the IVIS system. **(B)** Body weight was measured during drug treatment. **(C)** The lower panels show the results of quantitative analysis of tumor progression. ***p < 0.001 compared to the placebo group. **(D)** Levels of the PARP and cleaved caspase 3 proteins were determined by Western blotting. The lower panels show the results of quantitative protein analyses. *p < 0.05; **p < 0.01 compared to the control group.

T24 cells were implanted into null mice and allowed to grow for 1 month. Then, the animals were divided into four groups: the CTL, Hono, Mag, and Hono + Mag groups. After 20 days of drug treatment, the tumors in the drug-treated groups were inhibited compared to those in the CTL group (Figures 6A,C). The body weights in each treatment group were unaffected (Figure 6B). Moreover, cleaved caspase 3 levels were elevated in the Mag and Hono + Mag groups (Figure 6D). In addition, AKT phosphorylation was reduced in the Hono and Hono + Mag groups compared to the CTL group (Figure 6D). According to these results, Hono combined with Mag reduced bladder cancer progression *in vivo* through inducing apoptosis and inhibiting pAKT.

## Discussion

**FIGURE 7:**
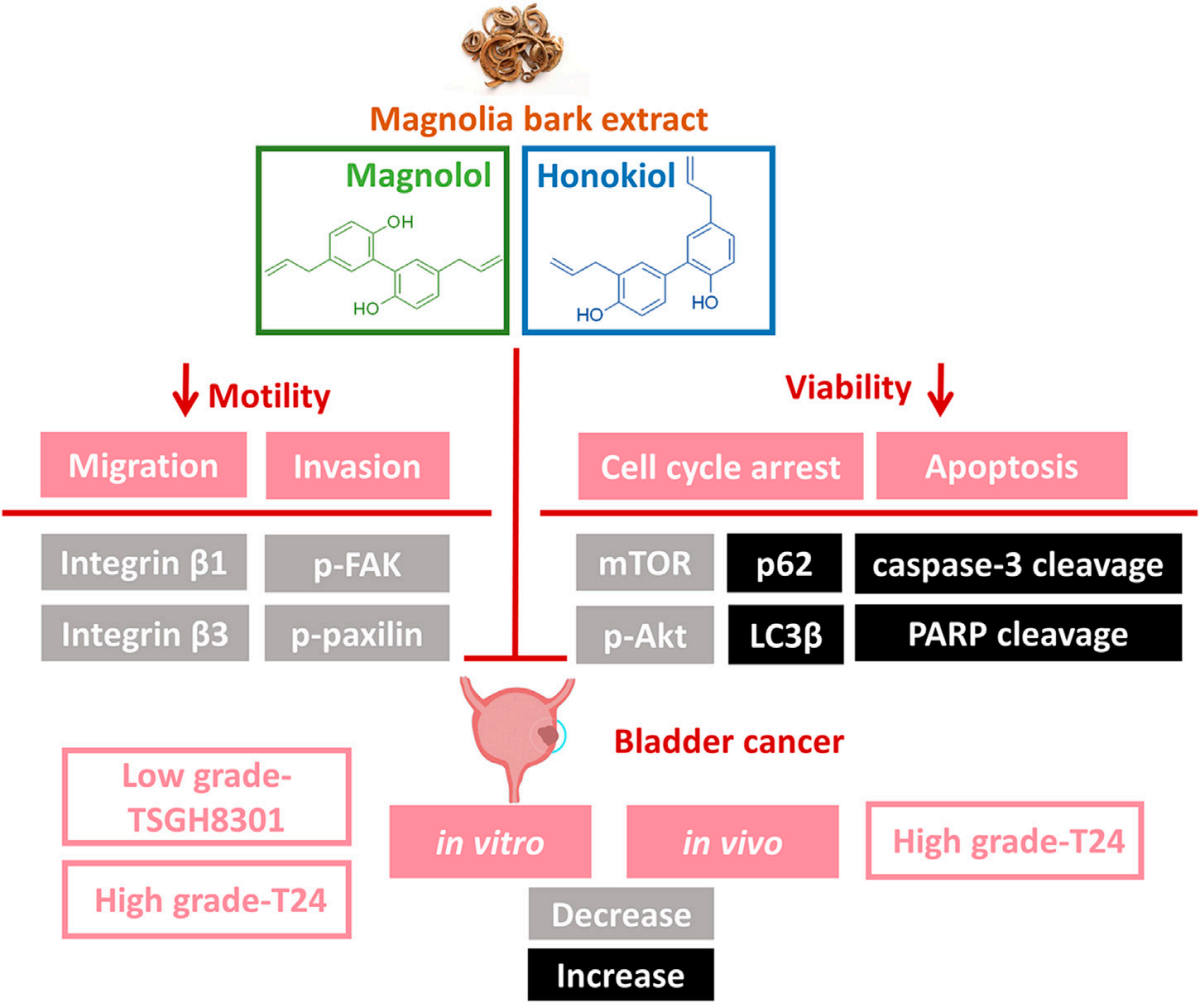
Illustration of Hono- and Mag-induced apoptosis and migration signaling pathways in bladder cancer cells. The combination of Hono and Mag caused cell cycle arrest and caspase-dependent apoptosis. In addition, Hono and Mag reduced bladder cancer migration through inhibition of the focal adhesive complex.

Through the use of high- and low-grade bladder cancer cell lines, we demonstrated that the combination of Hono and Mag amplified antineoplastic effects through apoptosis and autophagy regulation (Figure 7). However, the T24 and TSGH-8301 cell lines show several distinct characteristics. First, T24 cells were derived from a muscle-invasive tumor with poor differentiation, whereas TSGH-8301 cells are categorized as noninvasive and were derived from a low-grade tumor (Yeh et al., 1988; Zuiverloon et al., 2018). In addition, T24 cells were obtained from a Caucasian female, while TSGH-8301 cells were derived from an Asian male. Third, the TP53 gene status of T24 and TSGH-8301 cells is mutated and wild-type, respectively (Earl et al., 2015). Due to advances in understanding the pathogenesis of bladder cancer, it is recommended that the evaluation of drugs under development encompasses differences in pathological grade, muscle-invasive behavior, and ethnicity (Yee et al., 2011; Knowles and Hurst, 2015). Thus, the increased apoptosis observed in T24 and TSGH-8301 cells addresses the above factors and supports future investigation of combined therapy with Hono and Mag.

According to wound-healing and Transwell assays, inhibition of the migration of T24 and TSGH-8301 cells was identified exclusively in cells under combined treatment with Hono and Mag. Further examination of adhesion-related signaling revealed the downregulation of integrin β1, integrin β3, p-FAK, and p-paxillin. The integrin β subunit has been characterized as a therapeutic target due to its abundance in high-grade bladder cancer cells and correlation with mitomycin resistance (Zhang et al., 2012). The application of integrin β1 antibody was shown to attenuate adhesive and invasive behaviors in drug-resistant urothelial cancer cell lines (Vallo et al., 2017). In addition, integrin signal transduction was found to recruit FAK and paxillin, which are required for intracellular integrin signal transduction (López-Colomé et al., 2017). The targeting of FAK/paxillin pathways with some small-molecule drugs has been evaluated in bladder cancer studies with promising preliminary results (Chen et al., 2011; Chiu et al., 2016). Our results revealed the potency of the combination of Hono and Mag in the blockade of integrin-mediated cell adhesion. Future studies to investigate Hono-Mag-based therapy for metastasis prevention in bladder cancer are warranted.

The subcutaneous injection of T24 cells with Hono and Mag in combination had a tumor-suppressive effect without changing body weight. The adverse effect of weight loss severely hampers adherence to traditional chemotherapy in bladder cancer patients (Shariat et al., 2009).The efficacy of the nontoxic combination of Hono and Mag in bladder cancer was revealed by our *in vivo* results. Despite *in vitro* findings showing the anti-proliferative and anti-invasive effects of Hono and Mag, changes in *in vivo* bioluminescence imaging were too subtle to detect among all groups. The potency of Hono and Mag as adjuvants for chemotherapy has been established in the literature (Arora et al., 2012; Ranaware et al., 2018). As a result, one plausible explanation for our *in vivo* data could be the absence of common chemotherapeutic agents. A follow-up investigation should evaluate the strength of an increased dose of Hono and Mag in combination with chemotherapy in treating bladder cancer. Interestingly, tumor homogenates from the Hono and Mag combination group revealed decreased p-Akt protein levels, which was consistent with the *in vitro* results in T24 cells. Targeting the PI3K/AKT/mTOR pathway with small-molecule inhibitors is an emerging field in bladder cancer (Liu et al., 2018; Sathe and Nawroth, 2018). Our cellular and animal data may indicate the therapeutic mechanism of combination treatment with Hono and Mag through p-Akt suppression, which may be useful for future bladder cancer studies.

## Data Availability Statement

The raw data supporting the conclusions of this article will be made available by the authors, without undue reservation.

## Ethics Statement

The animal study was reviewed and approved by Laboratory Animal Center of the National Defense Medical Center, Taiwan (IACUC No. 19-229).

## Author Contributions

All authors have contributed significantly to this article. Writing—original draft preparation, H-HW; YC; C-YC; investigation, H-HC; P-JL; HM; Y-CC; writing—review and editing, YC; S-TW; project administration, H-HW; YC; funding acquisition, H-HW; S-TW; supervision, YC. All authors have read and agreed to the published version of the manuscript.

## Funding

This study was supported by grants from the Cheng Hsin General Hospital and National Defense Medical Center (CHNDMC-108-5 and CHNDMC-109-5), Tri-Service General Hospital (TSGH-E-109216), and Ministry of Science and Technology (MOST 107-2320-B-016-011-MY3).

## Conflict of Interest

The authors declare that the research was conducted in the absence of any commercial or financial relationships that could be construed as a potential conflict of interest.
